# Supplementary material for: Effect of 15 BMI-Associated Polymorphisms, Reported for Europeans, across Ethnicities and Degrees of Amerindian Ancestry in Mexican Children
Source: Int J Mol Sci. 2020 Jan 7;21(2):374. doi: 10.3390/ijms21020374 (PMC7013683; doi:10.3390/ijms21020374)
Supplement: Supplementary file 1 [file ijms-21-00374-s001.pdf]

Effect of 15 BMI-associated polymorphisms, reported for Europeans, across ethnicities and degrees of Amerindian ancestry in Mexican children.

Paula Costa-Urrutia[1,2] corresponding autor

E-mail: paula.costa.urrutia@gmail.com

[1]Laboratorio de Medicina Genómica del Hospital Regional Lic. Adolfo López Mateos, ISSSTE, Mexico City, Mexico.1321 Universidad Avenue P.C. 1321, Álvaro Obregón, Florida, 0103Mexico City, Mexico.

[2]Integrigen de Mexico SAPI de CV. Avenida Patriotismo 102-2. P.C 06100.

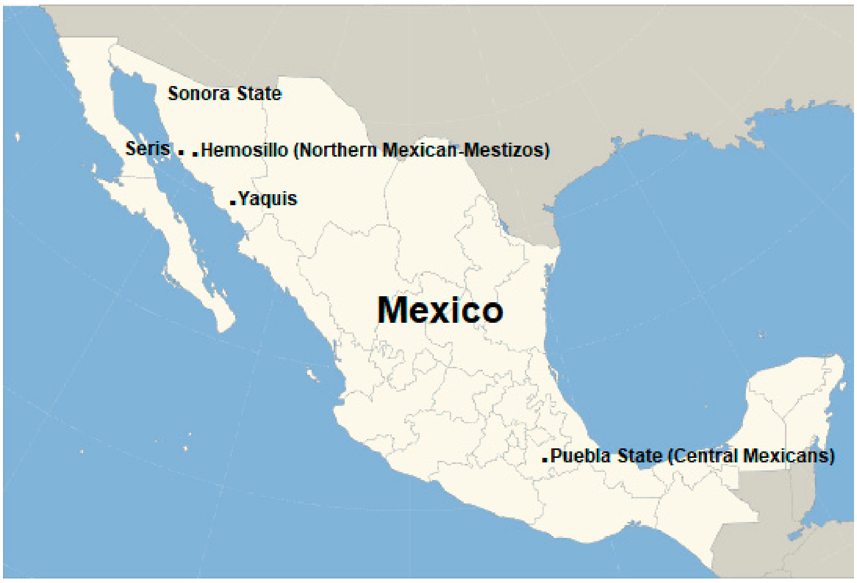


**Figure S1.** Locations of the different ethnic groups used in this study, Northern Mexican Mestizos from Hermosillo, Seris from Punta Chueca, Yaquis from Bahia de Lobos in Sonora State and Central Mexican children in Puebla State, Mexico.

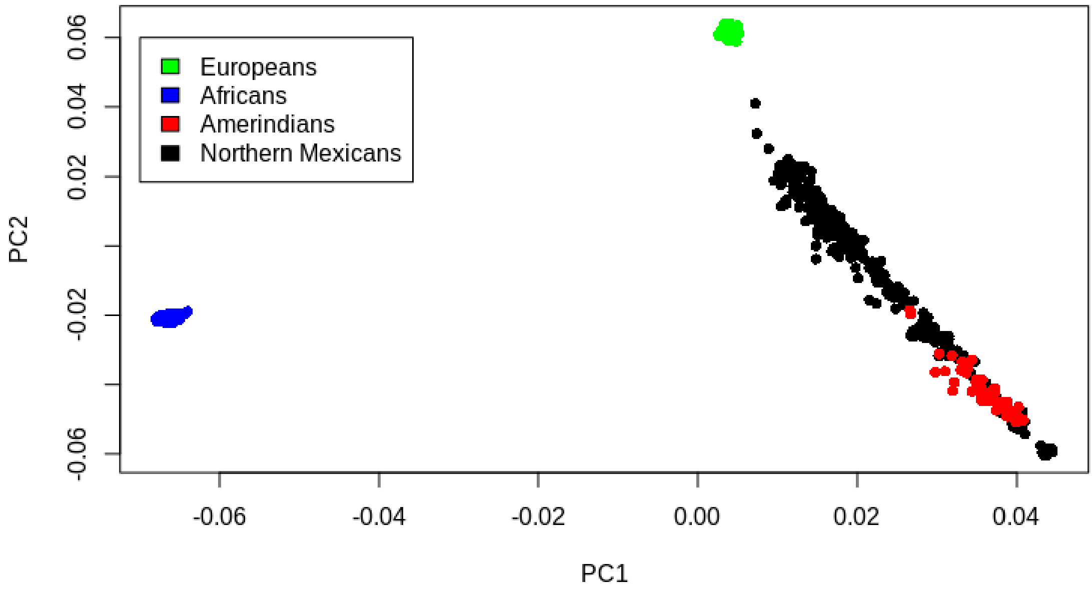


**Figure S2.** Fine-scale genetic stratification of the Northern Mexican children. Principal Component Analysis showed the two most informative eigenvectors (PC1 and PC2): a spread cluster which represents the Mexican-Mestizo and the Yaqui Amerindian ethnic group, and a separated tight cluster (lower right corner) which represents the Seri Amerindian ethnic group. The analysis included African, European, Amerindian, and Northern Mexican samples. The spread of each cluster is indicative of the level of relatedness in each population.

**Table S1.** General descriptive data of participants. The number of children (n), mean (m) standard deviation (SD) and minimum and maximum (Min-Max) of age, body mass index (BMI) and Amerindian ancestry (AMA) described in percentage (%) of each ethnic group (NMM: Northern Mexican Mestizos, CM (Central Mexicans).

| Ethnic group | Age | | BMI | | AMA(%) |
|---|---|---|---|---|---|
| | m(SD) | Min-Max | m(SD) | Min-Max | m(Min-Max) |
| NMM (n=178) | 7.6(2.2) | 4-12 | 18.9(4.4) | 12.0-33.4 | 43(17-83) |
| Yaquis (n=123) | 8.9(2.4) | 5-13 | 17.3(4.2) | 11.7-33.9 | 72(45-96) |
| Seris (n=68) | 9.5(1.7) | 5-12 | 18.6(4.4) | 12.9-39.3 | 92(56-99) |
| CM (n=8545) | 8.6(1.7) | 5-12 | 17.6(3.2) | 11.2-36.8 | 82(30-99) |

**Table S2**. Frequency of the associated allele in the 15 BMI/Obesity associated SNPs in Europeans, also associated in Northern Mexican-Mestizos (NMM), Yaquis, Seris and Central Mexican (CM) ethnic groups of children. (CHR: chromosome, A2 alternative allele).

| Gene | CHR | SNPs | Associated Allele | A2 | NMM | Yaquis | Seris | CM |
|---|---|---|---|---|---|---|---|---|
| *GPR61* | 1 | rs7550711 | T | C | 0.01 | 0.00 | 0.00 | 0.00 |
| *ADAM23* | 2 | rs13387838 | A | G | 0.03 | 0.01 | 0.00 | 0.00 |
| *MC4R* | 18 | rs6567160 | C | T | 0.10 | 0.07 | 0.05 | 0.05 |
| *LMX1B* | 9 | rs3829849 | A | G | 0.18 | 0.15 | 0.08 | 0.08 |
| *FTO* | 16 | rs9939609 | A | T | 0.24 | 0.09 | 0.09 | 0.12 |
| *FAIM2* | 12 | rs7132908 | A | G | 0.22 | 0.11 | 0.08 | 0.14 |
| *FAM120AOS* | 9 | rs944990 | A | G | 0.23 | 0.22 | 0.47 | 0.20 |
| *SEC16B* | 1 | rs543874 | G | A | 0.17 | 0.16 | 0.21 | 0.23 |
| *TNNI3K* | 1 | rs12041852 | A | G | 0.33 | 0.20 | 0.07 | 0.27 |
| *GNPDA2* | 4 | rs13130484 | T | C | 0.32 | 0.33 | 0.49 | 0.30 |
| *ELP3* | 8 | rs13253111 | G | A | 0.47 | 0.37 | 0.46 | 0.37 |
| *HOXB5* | 17 | rs9299 | A | G | 0.39 | 0.50 | 0.45 | 0.40 |
| *RAB27B* | 18 | rs8092503 | G | A | 0.30 | 0.44 | 0.15 | 0.41 |
| *OLFM4* | 13 | rs9568856 | G | A | 0.42 | 0.48 | 0.31 | 0.49 |
| *OLFM4* | 13 | rs12429545 | G | A | 0.35 | 0.44 | 0.32 | 0.49 |

Table S3. Power analysis by allele frequency (AF) and ethnic group; Northern Mexican-Mestizos (NMM)-Yaquis, Seris and Central Mexican (CM). β and OR represent (for linear and logistic model respectively) the minimum effect size that would be detected according to allele frequency, sample size and the parameters used (BMI mean=18.2, SD=4.3 and overweigh-obesity prevalence=34%) with an 80% of statistical power.

| | NMM-Yaquis (n=301) | Seris (n=68) | CM (n=8545) | NMM-Yaquis | Seris | CM |
|---|---|---|---|---|---|---|
| AF | β | β | β | OR | OR | OR |
| 0.05 | 2.00 | 4.00 | 0.40 | 1.70 | 3.70 | 1.10 |
| 0.10 | 1.00 | 3.00 | 0.30 | 1.50 | 2.60 | 1.10 |
| 0.15 | 1.50 | 2.70 | 0.26 | 1.45 | 2.30 | 1.05 |
| 0.20 | 1.30 | 2.50 | 0.23 | 1.40 | 2.07 | 1.07 |
| 0.25 | 1.25 | 2.30 | 0.21 | 1.40 | 2.00 | 1.07 |
| 0.30 | 0.95 | 2.00 | 0.20 | 1.35 | 1.90 | 1.06 |
| 0.35 | 0.93 | 2.00 | 0.19 | 1.33 | 1.85 | 1.06 |
| 0.40 | 0.90 | 1.70 | 0.19 | 1.33 | 1.85 | 1.06 |
| 0.45 | 0.88 | 1.70 | 0.19 | 1.30 | 1.80 | 1.06 |
| 0.50 | 0.86 | 1.70 | 0.18 | 1.30 | 1.80 | 1.05 |
